# Priscilla Idele: fertility, choice and changing norms

**DOI:** 10.2471/BLT.26.030226

**Published:** 2026-02-01

## Abstract

Priscilla Idele talks to Gary Humphreys about the drivers of falling fertility rates and the need for data-driven rights-based, youth-led responses.

*Q: The total fertility rate is declining in many countries. What do you see as the primary drivers of this trend?*

A: There are many dimensions to this, but let’s start with the facts: about two-thirds of countries now have fertility rates below the replacement level of 2.1 children per woman. This trend is most pronounced in high-income countries and parts of southern Asia, but it’s accelerating in many middle-income countries, and faster than the rate of decline seen historically in wealthier nations. As to what is driving it, the picture is complex. It is often discussed in terms of women not wanting to have babies due to changing aspirations and priorities, but UNFPA’s *State of the world population report 2025, The real fertility crisis: the pursuit of reproductive agency in a changing world*, revealed that around 1 in 5 of the 14 000 people surveyed across 14 countries reported being unable to have the number of children they would like to have, with over 50% citing economic barriers such as high cost of living, job insecurity and unaffordable housing.

*Q: To what extent is urbanization a factor?*

A: It’s clearly significant, but urbanization is also a complex phenomenon, since cities themselves are complex, generally comprising a wide range of socio-economic conditions and determinants. Often yielding benefits such as better education, health care and job opportunities, especially for women, cities also impose higher costs, limited space and greater competition for housing, childcare and education. In rural areas, children are often viewed as contributors to household labour and income. In cities, they are more likely to be seen as a financial burden. So urbanization fundamentally alters both the economic rationale and social expectations around family size. Migration also plays a significant role in driving down the total fertility rate, as indicated by the *State of the world population report 2025*. For example, outward migration is accelerating fertility decline in eastern Europe by shrinking the pool of young adults of reproductive age, especially women. Migration also disrupts family support networks and may introduce lower-fertility norms in host countries.

“There is no one-size-fits-all optimal fertility rate.”

*Q: What are the implications of the declining total fertility rate for sustainable development?*

A: Well, there too, the picture is mixed. It’s increasingly common to hear concerns about falling fertility rates, especially in wealthier countries with ageing populations. Fewer births mean fewer workers in the future, and more elderly people needing health care and social welfare. This can slow economic growth, because there are fewer working-age people to support the economy and pay taxes, and more retirees needing pensions and health care. But the story is different in many developing countries, where fertility rates are declining from previously high levels. In these contexts, a decline in fertility can be beneficial, creating a “demographic dividend” whereby countries with a larger proportion of working-age people relative to dependents (children and the elderly) can see higher productivity, greater economic growth, more resources for education, health and development. 

Declining fertility signals progress – greater gender equality, better education, better health care, and fewer maternal and child deaths. It can also reduce environmental stress. But it creates challenges too, like aging populations and a shrinking workforce. These challenges aren’t insurmountable but meeting them does require foresight. The real issue, I see, is that many governments have failed to anticipate and plan for demographic change. We’ve had population projections for decades, but policies have not evolved accordingly. Now we are having to adapt quite quickly, through major infrastructure, care systems, education and labour market reforms. 

*Q: Some governments have responded to these different development challenges and opportunities by developing pro-natalist or anti-natalist policies. What is your view on that?*

A: I believe that the goal of sustainable development should never be linked to achieving a specific fertility rate, whether this is in high-fertility or low-fertility countries. We need to be very clear: there is no one-size-fits-all optimal fertility rate. The idea that there is – whether it’s to grow the economy or preserve the environment – can easily veer into repressive, authoritarian territory. My position is that every individual should be able to make informed choices about reproduction, free from coercion, discrimination or fear. That means resisting both pro-natalist and anti-natalist pressures. 

In places where fertility rates remain high, you often hear policy makers talking about high fertility being a problem that needs to be solved. That framing is both disrespectful and ineffective. The real issue is not how many children a woman has, but whether she chooses to have them at a time she wants. In reality, many women in high-fertility contexts face limited choices. Early marriage, low access to contraception, lack of education, or societal pressure to have large families – all impose constraints. Having grown up in Kenya, I have personal experience of this. I was born in a rural town in western Kenya where large families were the norm and it was considered important to have a mix of boys and girls. My own marriage ended partly because I chose not to have more children. I had three daughters, and there was pressure from my in-laws that I try for a son. I was told, “Our son cannot die with only daughters. When is the next baby coming?” I did not want another baby. As a result, I experienced both physical and psychological violence from my in-laws. So I left. So, yes, when we talk about fertility, we have to talk about power, social norms, and autonomy. And this applies to low-fertility countries too. It could be argued that in low-fertility countries, social pressures discourage women from having children. In some countries, having children is somehow seen as opting out, choosing not to have a career, or not to fully participate in social life. Whether the pressure is to have children or not, the deeper issue is the same: people don’t feel truly free to choose when and how many children to have.

*Q: What’s your view of governments offering financial incentives to encourage women to have more babies?*

A: Governments should ask what people really want and not hand out baby bonuses. As shown in the *State of the world population report 2025*, narrowly focused pronatalist policies such as baby bonuses rarely work. Financial considerations are an issue, as the report itself shows, but the reality is far more complex. Governments need to address structural inequities that limit individual choices. That means investing in comprehensive sexual and reproductive health services, eliminating sexist norms that predominantly place the burden of child-rearing on women; and reforming laws that restrict bodily autonomy, implementing flexibility in the workplace, accessible childcare, and affordable housing. In the report, many women cited unequal sharing of domestic responsibilities as a key factor in not having babies. We need to change all that. We need to support reproductive agency through investments in gender-equitable caregiving, affordable fertility care, paid leave, and strong data systems. And develop policy by listening to the people most affected, and by that I mean young people.

“We need to support reproductive agency.”

*Q: What can be done to engage young people in developing policy in regard to sexuality and reproductive rights?*

A: We have to start by acknowledging that young people must be engaged – not just as subjects of policy, but as drivers of it. Their insights, innovations, and lived experiences are invaluable in shaping reproductive justice agendas. Too often, they are excluded from decision-making spaces or tokenized. To change that, we must create meaningful platforms for youth participation, fund their initiatives, and trust their leadership. That includes young people of all backgrounds and identities. At UNFPA, we work with young people as co-creators of solutions because we believe their voices and leadership are essential to achieving rights and choices for all. Through platforms like the Youth Advisory Panels in over 70 countries, young people help define national priorities and ensure that reproductive health policies reflect their realities. Initiatives like My Body, My Life, My World provide space for youth storytelling, where they share personal experiences that challenge stigma and amplify lived realities around sexuality and reproductive rights. We also support digital advocacy through programmes like the Youth against sexual violence and harmful practices campaign and collaborate with influencers and online platforms to reach young audiences where they are. In civic spaces, we partner with youth-led organizations to strengthen their capacity to engage in policy dialogues, monitor accountability and hold governments to their commitments, especially around access to comprehensive sexuality education and youth-friendly services. Across all these efforts, we’re seeing young people not just participating, but leading. They’re reframing the conversation around gender equality and reproductive rights, turning it into one that centres on dignity, agency and justice.

*Q: What keeps you awake at night?*

A: Data. We are facing a serious and growing data crisis. The defunding of the Demographic and Health Surveys, which have long been the backbone of global population and health data, is a profound setback. These surveys provided disaggregated, granular insights into fertility patterns, contraceptive use, maternal health and gender-based inequalities. Censuses and civil registration and vital statistics (CRVS) systems, while foundational, are not enough on their own. Censuses happen only every ten years, and CRVS systems, especially in low-resource settings, often lack completeness and consistency. Crucially, they don’t capture the deeper social and behavioural dimensions of reproductive health, like autonomy, access, or unmet need for contraception. Without regular, high-quality data, we can’t track trends, identify inequities, or evaluate whether our interventions are working. It means policy-makers are making decisions in the dark. We’re flying blind at a moment when clarity is most needed. If we are serious about rights and choices and sustainable development, we must treat investment in data systems as non-negotiable. It’s not just a technical issue, it’s a justice issue.

*Q: Do you worry about the global fertility rate?*

A**:** Not so much. I believe people still want to have children, and our UNFPA report supports that view. In the 1990s, we feared a population explosion; now we’re anxious about decline. The solution is not panic or knee-jerk reactions – it’s a matter of anticipating and planning for demographic change and building resilient systems that enable choices and rights for all, whether people want four children or none. Let’s support autonomy, equity and dignity. That’s how we will build a sustainable, inclusive society for current and future generations.

